# Network Analysis and Visualization of Mouse Retina Connectivity Data

**DOI:** 10.1371/journal.pone.0158626

**Published:** 2016-07-14

**Authors:** Bernard A. Pailthorpe

**Affiliations:** Brain Dynamics Group, School of Physics, University of Sydney, Sydney, NSW, 2006, Australia; Dalhousie University, CANADA

## Abstract

The largest available cellular level connectivity map, of a 0.1 mm sample of the mouse retina Inner Plexiform Layer, was analysed using network models and visualized using spectral graph layouts and observed cell coordinates. This allows key nodes in the network to be identified with retinal neurons. Their strongest synaptic links can trace pathways in the network, elucidating possible circuits. Modular decomposition of the network, by sampling signal flows over nodes and links using the InfoMap method, shows discrete modules of cone bipolar cells that form a tiled mosaic in the retinal plane. The highest flow nodes, calculated by InfoMap, proved to be the most useful landmarks for elucidating possible circuits. Their dominant links to high flow amacrine cells reveal possible circuits linking bipolar through to ganglion cells and show an Off-On discrimination between the Left-Right sections of the sample. Circuits suggested by this analysis confirm known roles for some cells and point to roles for others.

## Introduction

Detailed data on synaptic connections between nerve cells is becoming available now [1–3]. This provides hope that the structure of specific neural circuits can be determined and related to the underlying fabric of neurons and synaptic connections. Such could provide a platform for interpreting the available functional probes [4] and thus elucidate and eventually understand their functions within brain systems. Analysis of emerging connectomic data is anticipated to yield new understanding of retinal architecture and cell functions [5]. Network theory provides a framework for studying interconnected systems [6], comprising nodes, such as: an internet router, a person, an airport, etc; and their linkages (eg. a cable, a friendship or collaboration, an airplane route, etc). Applications have been wide ranging: from the internet, friendship and collaboration networks, transportation networks, ecological networks, through to metabolic and genomic networks. More recently network models are being applied to nerves and brains [7]. Network theory has a formal basis in mathematical graph theory [8, 9], so that many reliable results are available, along with good algorithms and computer codes in commonplace languages such as Matlab. Many methods rely on linear algebra, so that they are basically simple and often are scalable to very large systems–such as a brain. Visualization of networks via a computer graphics drawing can illustrate structure and functions as well as providing insights into information processing circuits. Already network theory and visualization [10] have been applied to the nervous system of the nematode worm, C. Elegans [1]. Further network studies have provided hierarchical, modular decompositions of that nervous system [11], which is expected to be an organising principle in brain networks [12].

Here we apply network analysis to the recently reported connectivity map of a 950 neuron sample of the mouse retina Inner Plexiform Layer [2]. Since this data covers a small sample of the retina it is not yet a connectome, but still is the largest and most detailed cellular level connectivity map available to date, and represents a significant step forward. The study aims to identify circuit features that might be consistent with the extensive knowledge of retinal function built up over the last century [13, 14]. This throws some light on a basic question: can network analysis of emerging connectomic data deliver biologically relevant insights into the function of the system under study? Partial success was achieved by using network metrics to identify key nodes and links, and combined with spatial information available from microscopy, to generate useful visualizations of possible information pathways and circuits in the retina.

## Methods

### Connectivity Data

The basic data used in this study is the cell-to-cell contact list for the inner plexiform layer (in the mouse retina [2], as determined by serial block-face electron microscopy (EM) of an approximately 0.1mm sized tissue sample. Specifically this is published with [2], which lists 579,725 possible cell-to-cell contact pairs, their observed contact area and x-y-z coordinates, within the 132 x 114 x 80 μm sample volume. An earlier study [15] identified synaptic vesicles and guided the discrimination of true synaptic from incidental contact areas. They found [2] a 50% probability of synaptic link at a 0.08 μm^2^ contact area (95% at 1 μm^2^). As in [2] we restrict the analysis to the 950 neurons in that volume and omit the 173 glia cells. 39 neurons were found to have no physical links, as defined above: of these five were rod Bipolar Cells (remainder not identified, but possibly Horizontal cells [2]). Examination of the EM images [2] confirmed that these neurons all were on, or very close to, the edge of the excised sample volume, so any possible links may have been to neurons outside the sample.

Given the significant effort required to produce connectomic data and the early stages of network analysis, it is worth noting here that some care is required in aggregating the primary cellular contact data to form a network link list. This is a key step in extracting reliable theoretical results from the experimental data. The structure of the primary contact area data [2], along with the sensitive relationship between synapse identification and contact area threshold, means that a careful filtering and summation of all contacts was required to ensure a reliable tally of links. Correct aggregation of listed i-to-j, along with j-to-i, contact areas thus adjusted for a possible 14% over-counting of links.

The link list was first filtered to eliminate contact areas less than 0.08 μm^2^ as unlikely to be associated with synapse formation [2,15]. That threshold yielded 236,020 putative synapses, or some 41% of all reported contacts. Of these 70,662 were between uniquely identified cell pairs (i, j), indicating numerous multiple contacts. Those multiple contact areas were summed for each cell pair to yield a total contact area, and hence weight, for each i-j link in the network. Correct aggregation of listed contacts yields 59,859 unique bi-directional links, slightly less than the figure above. A more conservative 0.16 μm^2^ contact area threshold for synapse formation was chosen, and proved necessary to extract robust results using network methods. This filtering resulted in 48,244 bi-directional links–that is, 11% of all possible links amongst 950 nodes. The link weight was calculated from the synaptic contact area as a multiple of the chosen threshold for synapse formation. This weighted link list was then transformed into a weighted adjacency matrix, the basic input to subsequent network analysis. The resultant weighted adjacency matrix is presented in S1 Data.

The links so derived are structural; functionality of the links needs to be inferred from other experiments and analyses. Synaptic direction was not reported [2], so all links are taken here to be bi-directional or symmetric. That said, much is known about retinal structure and function [13, 14], which can indicate a preferred direction of signal flow in some cases: eg. from bipolar cells towards ganglion cells.

Neurons were numbered sequentially and grouped by cell types as originally reported [2]. Neurons #1–36 are Ganglion Cells (GC); 37–226 are Near Field amacrine cells (NFac); 227–389 are Wide Field amicrine cells (WFac), amongst which 260–274 are Off type Starburst amacrine cells (Off-SAC) and 358–370 are On type Starburst amacrine cells (On-SAC); 390–696 are cone Bipolar Cells (cBC); 697–840 are rod Bipolar Cells (rBC); while 841–950 were identified [2] as one-of-a-kind types, some of which may be Horizontal Cells (H) [2]. GC and cBC sub-types were also identified [2].

Neuron cell positions (x, y, z coordinates) within the sample volume were inferred from the soma (cell body) Centre of Mass (CM) position which were extracted from centroids recorded in the EM pictures [2].

### Network analysis, visualisation and software

The system of 950 neurons and their 48K connections forms a weighted network of linked nodes that can be studied by standard graph theory [8,9] and network analysis [6,7] techniques. A challenge for system of this type is to identify reliably what system components form the nodes and the links in the network. This has been discussed extensively in the context of brain imaging [16] and network analysis [17], especially in regard to distinguishing, or correlating, structural and functional links. While synaptic contacts inferred from EM may be more reliable in this regard than MRI based brain studies [18], where nodes are brain regions and links are derived from signal correlations, the present analysis shows that care must be exercised. Here a node is taken as a neuron, comprising a soma (cell body), which may be spatially extended but whose centroid location is identified in the EM pictures [2], along with dendrites and axons that usually are spatially extended, possibly over large distances. The definition of a link devolves on the extensive treatment of contact areas and synapse formation in the original EM studies [2, 15]. The multiple synapses to an individual neuron often are spatially dispersed, possibly reflecting distributed functions such as electric potential integration or chemical transmitter detection. Despite these considerations a network model and graph representation approximates each node as a point and each link as a single weighted line.

This network, or graph, is fully specified by the weighted adjacency, or connectivity, matrix whose elements A(i,j) are the strength of the i-j synaptic link between neurons i and j. Synaptic direction was not reported, so all links are taken here to be bi-directional, resulting in a symmetric adjacency matrix, ie. A(i,j) = A(j,i). Note also that some network analysis uses only the un-weighted adjacency matrix in which A(i,j) = 1 if a link exists between neurons i and j, and = 0 otherwise.

Numerous quantities characterise network structure and performance [6–7, 16–17]. Amongst these, measures of centrality which can indicate the importance of a node or link, of module or community structure, and of information flows are most likely to yield biologically relevant insights into retinal functions. Herein, node Degree (k), node Betweenness Centrality (nBC) and edge BC (eBC) are reported.

The Degree k _i_ of a node i is the number of edges connected to that node, and is calculated from the adjacency matrix: k_i_ = Σ_j_ A(i,j). In the present analysis all links are symmetric so that the in-degree and out-degree are equal. The Centrality measures count the number of shortest paths in the network that traverse a node (nBC) or a link (eBC). Thus they point to key nodes or links that may be traversed by traffic flows in the network.

### Network decomposition into Modules

It is widely expected that neural systems have a hierarchical, modular structure [7, 12]. Network methods offer one route to decomposing a network into its component modules. Various methods are available to calculate such decompositions [19]: spectral methods [6, 19, 20], such as clustering (eg. via k-means) of the eigenvectors of the graph Laplacian [8,9], or of the modularity matrix [21]; and by the Newman-Girvan agglomerative or divisive methods [22, 23], in which links are successively added or pruned to form sub-networks. One variant is the so-called fast Newman agglomerative algorithm [24, 25], with stability optimisation [26]. All these methods seek to maximise some modularity metric, such as Newman’s Q [20–23], which measures the fraction of within-module links compared to that expected for a random network with the same degree distribution. Thus the random graph, with Q = 0, is taken as a null model that should not have any clustering tendency. Higher Q (eg. Q > 0.4) may indicate good partitions, while small or near zero Q values indicate poor decompositions or a weak tendency to form modules in the network.

Given that a primary purpose of neurons is to transmit signals over the network of connections and to process information, it seems likely that measures related to information or signal flows on the network may lead to insights regarding possible neuron circuits. The InfoMap [27–30] algorithm performs a modular decomposition of the network by mapping the probability flows that the network structure induces, specifically by launching a large number of random walks onto the network of nodes and their weighted links. A monte carlo like sampling of these walks then estimates the fraction of signal flows through each node and link. Modules emerge as regions of the network in which the random walker has proportionally larger residence times. The random walk trajectories are described using signal coding theory [27, 30], specifically a Huffman code. This measures the information cost of describing the random walk trajectory on any modular partition of the network. A search for the most efficient, or minimum code length, yields the modular partition of the network. Such a process can serve as a proxy for information flows on the network which, for a nervous system, is likely to more appropriately characterise network functions over the available structure. Tests of the InfoMap method showed that it produced reliable decompositions of a variety of real-world networks [27]. Thus we adopted the InfoMap method herein. It is available as a set of C^++^ codes [30] that can be compiled locally on a desktop computer.

### Network visualization

A number of algorithms are available to layout and draw a graph of connected nodes in 2D or 3D graphics [31, 32]. Broadly, they are based on: the graph spectra [33] of the adjacency matrix A, of various graph Laplacians, or of graph paths; or separately on a force balance equilibrium of nodes connected by virtual springs [32]. The choice of visualization method reflects a balance of: its formal basis and reliability, scalability to very large systems, visual attractiveness/aesthetics [34], and utility of the final picture in gaining an understanding the system under study. The spectral methods have a sound mathematical basis and are scalable to large numbers of nodes, but often produce poor layouts that can appear crowded thereby inhibiting insight. The force balance techniques can produce attractive layouts but are based on an ad-hoc method that are not expected to be scalable, and here did not produce biologically relevant insights.

Several of the above methods were tested on the retina connectivity data, but most yielded visualizations of the retinal network that were too crowded and dense to be useful. Here we adopt the Spectral Distance Embedding (SDE) method [35] that performs a global optimization of geometric distances (in the 3D computer graphics representation) to the graph theoretic distances (ie number of hops over network links). Mathematically the graphics layout is specified by ||x_i_−x_j_ || ~ D _ij_ for all i-j pairs, where x _j_ is the location of a node in the computer graphics visualization, and D_ij_ is the shortest path length between any pair of nodes i and j in the network. This produces 3D graphics that appear to have a reasonable layout, while balancing crowding of nodes and edges with feature discrimination, such as highlighting key functional nodes.

Any plot of all 950 neurons and their 48K links would be so dense that little could be learned from it. A plot of all links shows a continuum fabric from which it is difficult to identify key neurons or circuits. Thus a strategy to identify landmark nodes and links is required, so that key features of the network can be uncovered. Various strategies were adopted, such as examining the highest Degree nodes, the highest weight links, the highest nBC nodes, and the highest eBC links. Finally, the InfoMap calculations that identify the nodes and links with highest signal or information flows, was adopted herein to identify the key nodes and links.

All calculations and visualizations were performed using Matlab (R2014a) codes, written and tested by the author [36], along with community code libraries to calculate standard network properties [37–40]. These visualization were also performed using Matlab codes which permit 3D rotation and zooming of the graphics, and were used to produce the figures included herein. Accuracy and performance of codes used were verified using simple test cases.

## Results

### Basic network features

The analysis begins by examining network connectivity measures since these provide an overview of structural features of the neuron network. Three basic measures are summarised in S1 and S3 Tables; these are node Degree (k), node Betweeness Centrality (nBC), and link or edge Betweenness Centrality (eBC). The Degree distribution of the weighted network of 950 neurons in the mouse retina sample is shown in S1 Fig as a log-log plot. While this looks suspiciously like a power law, no conclusions can be drawn. The data exhibit differing slopes for low and high degrees, where each covers less than 2 decades of Degree, indicating a lack of statistical support [41].

The highest Degree nodes (S1 Table), of the un-weighted network sums the number of synaptic links incident on a given neuron, while for the weighted network this sum also tallies the weight of each link (as indicated by the measured synaptic contact areas [2]). The lists are dominated by Ganglion Cells, with one putative Horizontal cell included in the top 10. Thus Degree was not a useful guide to network function. The highest nBC (S2 Table), calculated on the weighted network, lists the nodes traversed by the most network paths. Here the top entries are amacrine cells (ac), particularly those of starburst type, SAC-Off; and now also include a Direction Selective Ganglion Cells (DSGC). Overall, amongst all the neurons reported, the wide field ac, then DSGC and then putative Horizontal cells exhibit high values of nBC (S2 Fig). This is consistent with the functional roles of these cells [3, 4]. Given that the retina sample was reported [2] to contain elements of a direction selective circuit, nBC may be a better indicator of key functional nodes. Similarly the highest eBC (S3 Table), calculated on the weighted network, lists the network edges, or synaptic links, traversed by the most network paths. The most traversed links connect amacrine cells to rod Bipolar Cells (rBC).

Nodes in this retina network are closely connected. Calculation of all shortest paths (over the unweighted network) reveals 829K shortest paths connecting the 950 nodes. Of these, 84% comprise 2 steps, 12% are one step, and 4% are 3 steps.

The 0.1mm sample of 950 neurons under study represents a small fraction of the retina. Thus edge effects will arise since links will be lost during the sample excision procedure. This will most affect those neurons (eg. Horizontal Cells) with much longer range connections; many other neurons have shorter range connections, as seen in the EM pictures [2]. The scale of such effects is now illustrated. Possible edge effects might be evident in, for example, the outer 10% of the 80 x 114 μm sample (in the retinal plane). For simplicity sampled neurons, or network nodes, within 10 μm of those four edges were classed as edge. Those nodes located more than 10 μm from an edge were classed as core, and generally less likely to be affected by the edges. Core nodes had an average Degree of 865, compared to 679 for edge nodes, and 786 for the whole sample. For node Betweenness Centrality, core nodes had an average nBC of 1578, compared to 1174 for edge nodes, and 1375 for the whole sample. In each case there is a 20–25% effect on typical aggregate network measures, presumably due to sample excision induced edges in the network. In regard to the 39 neurons exhibiting no links (zero Degree), a cursory examination of the EM photos revealed that they were at or near the sample boundary, indicating that any links were likely excised.

### Network visualization

Any plot of all 950 neurons and their 48K links would be so dense that little could be learned from it. A plot of all links shows a continuum fabric in which it is difficult to identify key neurons or circuits. Thus a method to identify landmark nodes and links is required, so that key features of the network can be uncovered. Various strategies were adopted, such as examining the highest Degree nodes, the highest weight links, the highest nBC nodes, and the highest eBC links. The initial search for and analysis of possible circuits began by focussing on the most highly connected (highest Degree) nodes and the highest weight links. The latter have been shown in mouse visual cortex to link functionally correlated neurons [42]. The highest nBC nodes, and the highest eBC links all were studied and visualized. These did not reveal circuits or pathways that were credibly consistent with textbook knowledge of the retina [3, 4].

A typical network visualization is shown in S3 Fig which displays the network layout using the SDE method, highlighting the highest degree nodes (cf. S1 Table) and their high weight (>10) links, so that 1164 links are drawn, of the 4092 that exits linking these 10 nodes to all others.

This purely structural network analysis and visualization place a Horizontal cell as a key gateway to numerous GC’s; a result in contrast to others presented below. Centrality measures are likely a better indicator of gateway cells. S4 Fig shows the SDE network layout highlighting the 10 highest nBC nodes (cf. S2 Table) and their high weight (>10) links, so that 546 links are drawn, of the 3275 links between these 10 nodes and all others. This view now highlights more SAC-Off, but no SAC-On, and still includes many GC’s.

Several of the other network layout methods reviewed above were tested, but they yielded visualizations of the retinal network that were too crowded and dense to be useful. Ultimately, a network analysis combined with geometric locations, derived from EM photos, proved more useful, as presented below.

### Network Modules

Modular decompositions of the retina network were calculated using: k-means clustering of the eigenvectors of the graph Laplacian, and of the modularity matrix; and by the Newman-Girvan agglomerative and divisive methods listed above. Overall these methods produced variable and inconclusive results for the mouse retina network, and network visualizations using these approaches did not generate useful insights into retinal circuits. These decompositions were characterised by Newman’s modularity metric, Q. The results of the methods listed are summarised in S4 Table, with Q values typically ~ 0–0.3, indicating a weak/marginal modularity separation in the present sample. The most straightforward method, k-means decomposition of the graph Laplacian, produced its optimal Q value for 8 modules, but showed poor discrimination of neurons in the EM x-y plane. Other modularity detection methods produced varying results, sometimes combining modules shown above and/or yielding Q values, indicating poor modular decomposition.

The InfoMap modular decomposition is shown in Table 1. There are 10 modules of non-trivial size, meaning that each contains more than 15 neurons or hosts more than 1% of the signal flows. Of these 8 appear to be significant, with two of those containing the SAC On and Off cells. 28 notional modules contain only one neuron that appears to be a dangling node (with no apparent links), mostly these are putative Horizontal cells. They possibly have links beyond the 0.1mm sample size that were not captured [2]. The top 4 modules, which contain 72% of all signal flows, have respectively 200, 145, 168, and 153 member neurons. Module 7 contains all the SAC-Off neurons, while Module 8 contains all the SAC-On neurons. The top flow ac’s are listed in S5 Table. Sensitivity of the InfoMap decomposition to the contact area cutoff, corresponding to a unit weight link, was investigated. The Participation coefficient [43] was found to be large (P_i_ >0.9) for all nodes, indicating that between-module links predominate over within module links.

**Table 1:** Modular decomposition.

Module #	Total Flow	Number of neurons (cells)	key neurons	Color, used in figures
1	0.205	200	GC, NFac	red
2	0.191	145	GC, WFac	salmon
3	0.177	168	GC, WFac	blue
4	0.176	143	GC, NFac	green
5	0.091	92	GC, NFac	brown
6	0.047	67	NFac	light blue
7	0.034	23	SAC-Off	light purple
8	0.023	14	SAC-On	plum
9	0.022	15	WFac, H	grey
10	0.015	16	BC, H	grey
11	0.009	3	na	grey
12	0.006	10	WFac	grey

InfoMap decomposition of the network of 940 retina neurons. For each module is listed: the total fraction of signal Flow for all nodes within the module, the number of member nodes (neurons), the leading member types, and the color used to denote each module in the figures. Total signal Flow in the module is listed in decreasing order. Modules 1–4 also contain numerous BCs, of both cone and rod type. Colors used in the figures are listed.

**Fig 1:**
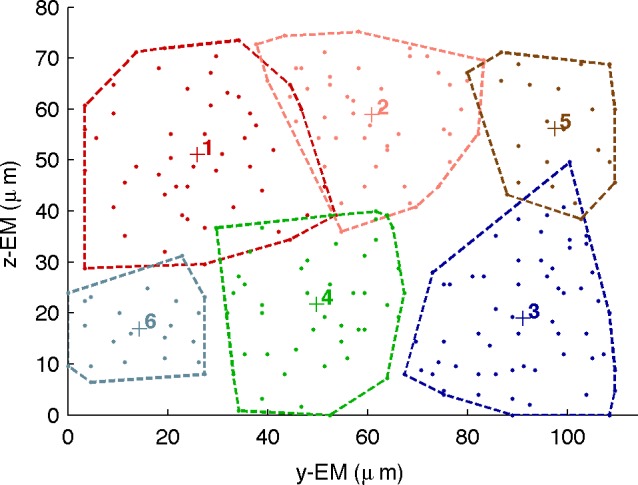
2D layout of cone BC modules in the mouse retina sample. EM coordinates in the 2D retinal plane of key cone BCs coloured by their module membership, along with the convex hull enscribing members of each module. The centroid location of each module is marked (+).

Fig 1 clearly shows a mosaic tiling of the cone BCs in the retinal plane when grouped by the InfoMap modules. Note that not all of the 306 cBC’s are shown, rather just those exhibiting high weight links to the SAC and key ac analysed below. The apparent gap on the right was investigated, to confirm that no relevant cBC’s were omitted.

Contrasting modular decompositions, such as the Newman agglomerative algorithm [22, 24, 25] produced broadly consistent results, but differ in details. An example is the cone BC module layout [in the EM plane shown in S5 Fig. Note that the InfoMap module 4 (shown as green) is missing, having been subsumed into module 3 (blue), which now extends over a larger retinal area; while other details, such as links routing via A2, are now changed (not shown). However the overall left-right diagonal partition in the retinal plane is preserved. This increases confidence in the results presented since it is predicted by two different methods. Decompositions by some of the other methods reviewed above did not produce sensible results. Each method produces a different ranking of nodes (S6 Table), indicating that other functionally relevant information needs to inform the analysis.

**Fig 2:**
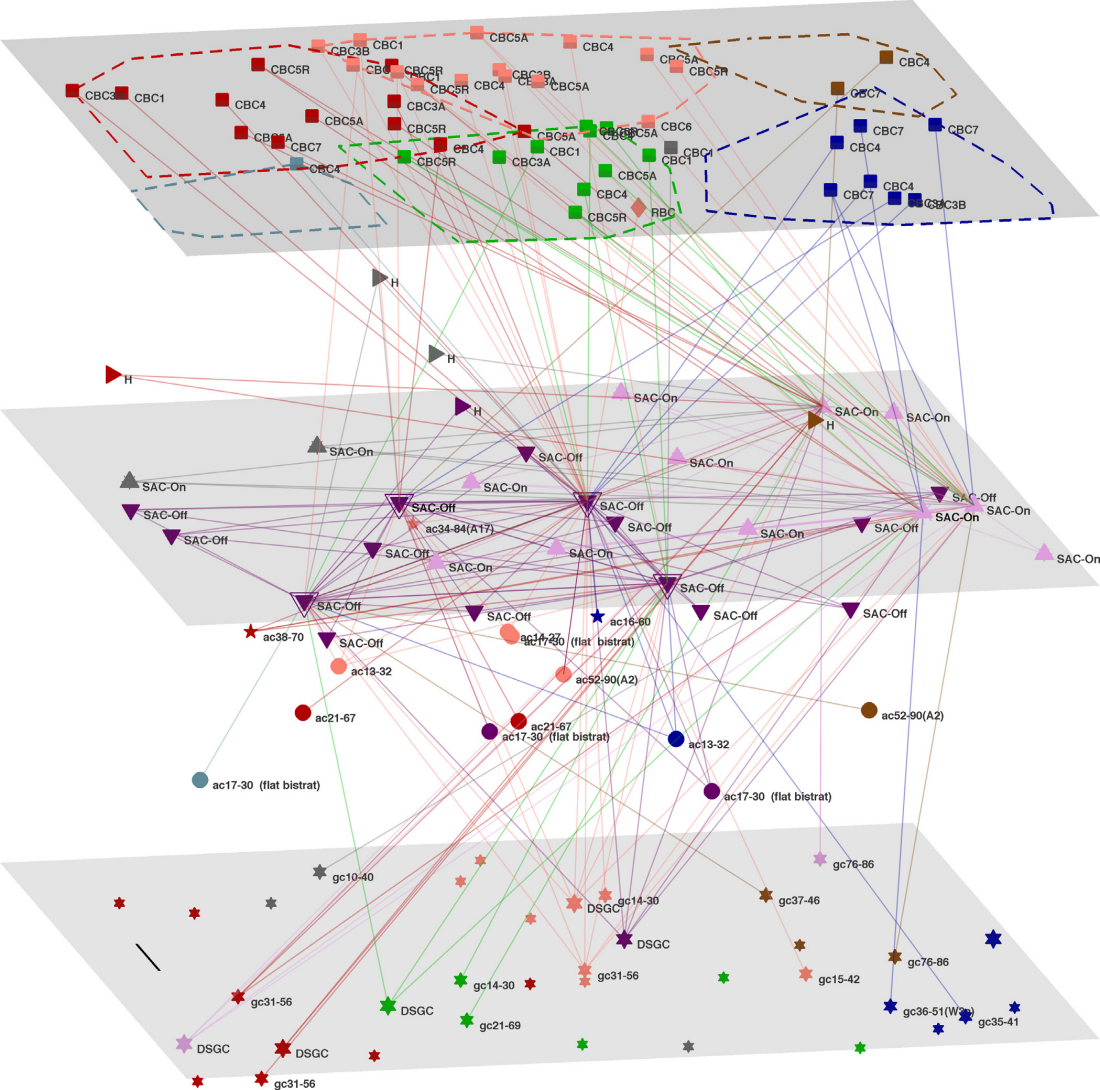
Layout of high signal flow SAC. 3D plot of SAC-Off and SAC-On cells, along with their highest weight (>10) links to all other neurons. Cell positions (in x-y plane), were calculated from the centroid of the soma in the EM photos [2]. Layers in the EM plane are schematic, estimated from the EM photos and standard texts [13, 14]. Scale bar, 10 μm. Module membership is color coded as in Fig 1.

Fig 2 reveals a separate fabric of links involving the SAC’s. Off-SACs dominate the LHS of the sample, while On-SACs predominate on the right. Even this fabric, filtered for the highest signal flow nodes, and strongest weight links, is complex. It shows a predominant left to right flow from CBC’s to SAC’s, and then right to left from SAC’s to GC’s. The circuits can be disentangled by individually examining the top SAC’s of Off- and On- types.

The highest flow of these is via a central Off-SAC (#260 [2]), shown in S6 Fig. It receives numerous inputs from cBC1, 3A, 3B & 4 on the LHS, along with 3 links from the RHS (CBC3B & 4). Its strongest link (weight 128), presumably outwards, is to a central A2 amacrine cell; there are weaker links to 4 other ac’s. This Off-SAC has 2 direct links (weights ~20, as derived from contact area) to central DSGC’s, along with weaker links to 5 other GCs, located centrally or to the right. The next Off-SAC, with the second highest flow, is on the LHS and shows a similar fabric of links. It is not shown since it reveals no additional insight.

The top On-SAC (#364) is located on the upper RHS of the retinal plane S7 Fig, and receives inputs from cBC5R on the left and centre, along with CBC7 which are on the left and right. Links of type 5 cBC’s to On- cell circuits have previously been observed [44]. Here the five links to types 5A and 5R, and also to type 7, are all relatively weak (weights 10–20). There are no strong links to rBC’s. This On-SAC has strong onwards links (weight 49, 17) to two DSGC’s on the LHS, along with a weak link to a central DSGC. There are links to 3 other GC’s. The next On-SAC (#358) also is located nearby on the RHS of the EM plane, as shown in S8 Fig. It predominantly draws links from the two centre modules (colored salmon and green in Fig 2) containing cBC’s of types 5A, 5R & 6 and otherwise has a similar fabric of links. There are no strong links to rBC’s. Note that these two On-SAC’s are linked by two putative H cells. Since this system is thought to be associated with an edge detector [2], one could speculate that these two On-SAC’s sequentially detect light differences that moves progressively from left to right across the retinal plane, and then signal to the DSGC’s that are laid out from right to left—ie. in the opposite direction.

**Fig 3:**
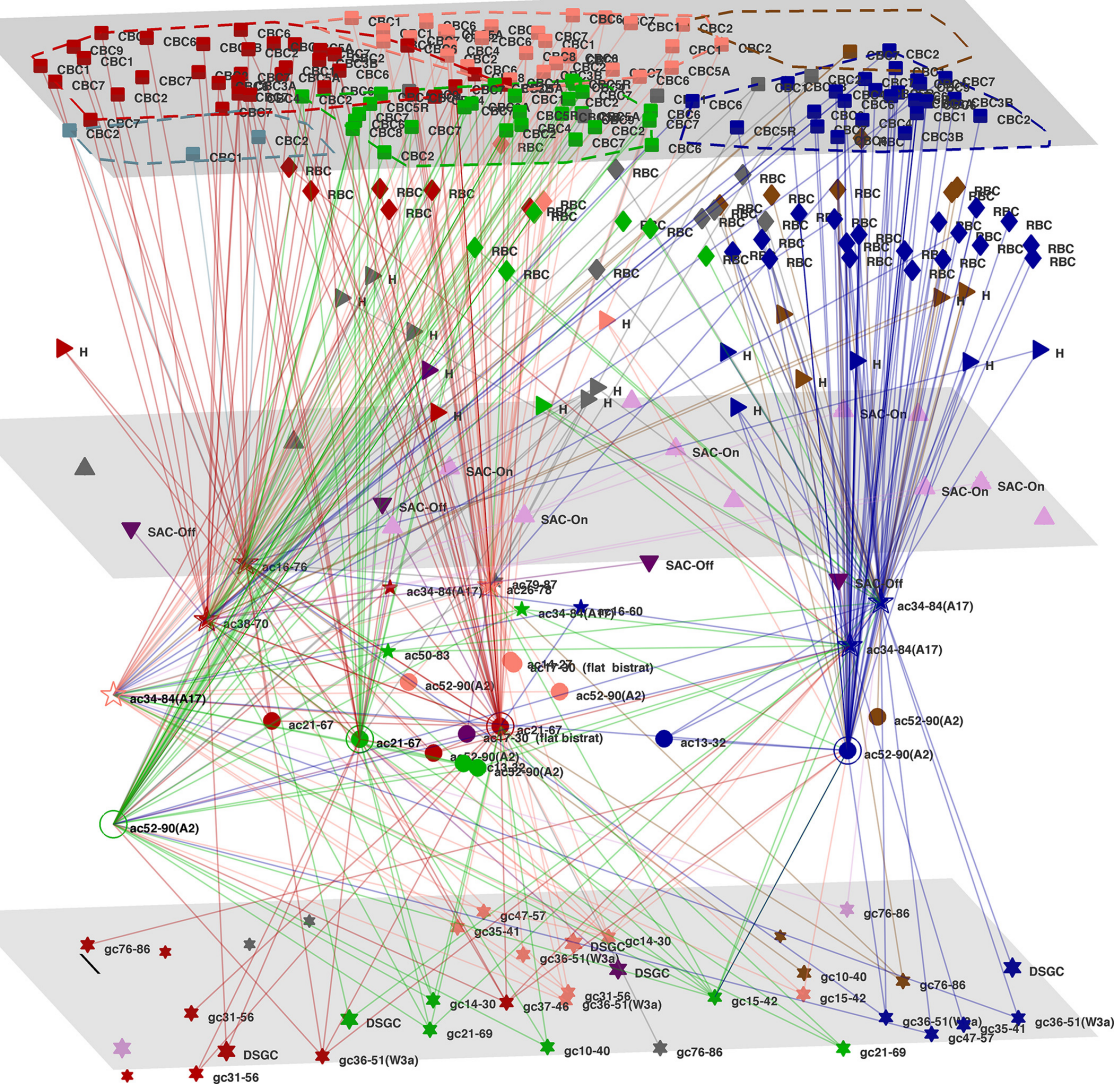
Layout of high signal flow amacrine cells. 3D plot of the top 10 amacrine cells, along with their highest weight (>10) links to all other neurons. Cell position (in y, z axes), were calculated from the centroid of the soma as revealed in EM photos [2]. Layers estimated as in Fig 2. Scale bar, 10 μm. Modules colored as in Figs 1 and 2.

Fig 3 reveals a few key linkages to the top amacrine cells, in 3 modules that are separated geometrically in the retinal sample plane (cf. Fig 1). In the module to the lower right (blue, module #3) one A2 and two A17 cells are the central hubs. The A2 cell shown receives 6 strong links from cone BCs, mostly Off type, with the strongest link (weight 200) from cBC-1; A2 also receives 7 strong links from rod BCs, with two very strong (weights 124, 156)); A2 has a strong onward link (weight 140) to a single DSGC; the two A17 cells have generally weak links to cone BCs and to most GCs, aside from a single strong link to a type W3a Ganglion Cell; and the two A17’s together receive numerous links from rod BCs, of which 9 are medium strength (weights >40). The module in the central top region (brown, #2) has two A2 and one A17 The other large module, to the left (red, #1) has amacrine cells of type ac21-67 and ac38-70 as its major signal hubs. Another smaller module on the left (green, #4) contains ac21-67.

The top flow amacrine cell (#120 [2], of type ac21-67) is located centrally in the retinal plane, and receives inputs predominantly from its own module in the upper right hand side (RHS), as shown in S9 Fig. Its strongest link, presumably inwards, is from two cBC-7 (weights 112, 78, and a cBC-6 (weight 38). Numerous other cBC’s, of types 1, 2, 3B, 4, 5A, 6 & 7 provide inputs, with 7 links from Off-type cBC’s and 11 from On-types. It also has inputs from seven rod BC’s, two with strong links (weights 85, 90). There are links to 6 ac’s and one Off-SAC. This top ac then links on to numerous GC’s distributed across the retinal plane. These include a central DSGC and a W3a to the left, both with relatively weak links. There have been suggestions [5, 45] that key ac can provide feedback signals to cBC’s or rBC’s. Presumably such would be weak (eg. weight 1 or 2) links, since their supposed function is gain control, and a general principle of stable feedback is that return signals should be relatively weak. Analysis of the data indicates that this ac has such links to cBC- 3A, 3B, 5R and the newly reported [2] type XBC, with none to rBC.

The next ac (#202 [2], of type A2, ac52-90) is located on the lower RHS in the retinal plane, and receives inputs predominantly from its own module, as shown in S10 Fig. Eleven links are from Off-type cBC’s, while 7 are from On-type. Its strongest link (weight 201) is from a local cBC-1, and then from two rod BC’s. Other inputs are from cBC-2, 3B, 4, 5R, 6, 7 & 9, all in the RHS module (#3, blue). Overall the On-type cBC links have stronger (30–200) weight, while the Off- links are weaker (weights 10–10), aside from one cBC-4 (#637) with weight 40. This A2 links to two A17 type ac’s. Its strongest onwards links (weight 119) are to a gc15-42 to the left, and to a type W3a GC locally (weight 25); other links are to 4 GC’s distributed across the retinal plane. Weak (eg. weight 1,2) possible feedback links to cBC’s from this ac are to cBC-2, 3A and 5X. The top flow A17 (#351 [2], or type ac34-84) is located on the RHS of the retinal plane (cf. Fig 3). It has possible feedback links (ie. weight 1,2) to 18 cBC’s (not shown), but only to two rBC’s.

Aside from results noted above, cBC-6 to -9 did not emerge with significant roles in this analysis, however their connections can be studied by these methods. For example, the seven cBC-9 neurons have 82 strong (weight>10) links to other neurons, of which 7 are very strong (>30), predominantly to ac and GC, and to one H.

In contrast to the above results, different methods for decomposing the network into modules and determining key ac’s produced less clear results. Most of the other techniques rely upon network structural measures, rather than signal flows as used above. For instance, the fast Newman agglomerative method [21, 22, 24] yields 4 overlapping modules in the EM plane. If the key ac’s are chosen from the top edge BC measures (S3 Table, column 1) then an A17 cell is the key gateway on the lower left of the EM plane, while an ac16-60 is key in the centre of the plane. Using node BC (S2 Table) as a measure to select the top ac’s was less informative and no clear picture emerged.

## Discussion and Conclusions

The first mouse retina IPL connectivity map was modelled as a network of nodes, representing neurons, and weighted links representing aggregated synaptic connections. The primary data is cell-cell contact areas observed by EM [2]. This is used to infer the network of links as described by an adjacency matrix, with synaptic contact areas used to calculate the network link weights. While this assumed area-weight correlation is not definite, the original analysis of cBC-GC contacts (see figure 4d in [2]), shows that our contact area cutoff of 0.16 μm^2^ indicates 2–3 synaptic contacts, and their observation that stronger connections are unlikely to be spurious further supports this approach.

The small sample size means that edge effects were introduced at the network edges, where links were likely deleted by the sample excision process. Indications are that this decreases typical network measures by at least 20% in regions adjacent to the sample edges. Neurons showing no links were mostly near the sample edges. Such matters can only be fully understood when larger samples become technically feasible.

Basic network analysis and graph visualizations, on their own, did not reveal useful insights into retinal circuits. Even with a focus on only the top nodes and their strongest links (eg. S2 Fig) it was not possible to extract meaningful insights from such plots, consistent with textbook understanding [13, 14]. Thus Degree, and other basic structural measures, alone, may not yield insights into function in networks of this type. Additionally, plots of graph spectra may appear unfamiliar and initially uninformative for biologists, much as a Fourier transform of an image or audio stream can take some getting used to before they generate useful insights. This highlights the need to identify and prioritize landmark nodes and links in the network, to guide further analysis. Network features overlaid on the geometric layout of neurons, measured from the EM images, proved to be more useful in delineating possible circuits.

Modular decomposition based on signal or information flows on the network more clearly identified biologically relevant nodes and links, whose connections could be further analysed. The InfoMap method, which follows random walks on many possible paths in the network is more likely to reflect dynamical aspects of the network of linkages and so may reveal functional information. For the retina network this proved to be more useful than analysis based on purely structural features of the network. This decomposition identified discrete modules of cone bipolar cells that form a tiled mosaic in the retinal plane (Fig 1). Such a 2D layout of modules, containing 10’s to 100’s of neurons, is reminiscent of that observed for cones at other scales in the retina [46]. The corresponding modules of ganglion cells have a similar tiled layout (not shown). Overall a modular decomposition based on signal flow produced the clearest picture in the present study, compared to other structural measures of network properties.

Key amacrine cells (A2, A17) and SAC’s are thus found to have the highest signal flows of all cells in the sample volume. This is consistent with their known roles [13, 14] as key gateways in the retina. Dominant network links to the highest flow amacrine cells reveal possible circuits and show an Off-On discrimination between the Left-Right sections of the sample retinal plane. Other amacrine cells (eg. ac21-67, ac38-70) are also highlighted as being key gateways for signals from cBC to GC. Once these key cells are identified then their link weights can be interrogated from the adjacency matrix, possibly suggesting important partners.

The combined visualizations presented herein, in which network modules are laid out geometrically in the retinal plane using observed EM coordinates, proved to be more useful. The six leading modules tile the retinal plane in a mosaic pattern, while two other modules contain all the SAC-On and SAC-Off cells and span the plane of the retinal sample. Taken together, these results suggest that an edge detector system, segregated by Off/ On functions in the Left/ Right regions, constitute a key feature of this retinal sample.

The results confirm central roles for cone Bipolar Cells: dominant links to Off-SAC are from cBC-1 to 4, while cBC-5A, 5R and 7 have strong links predominantly to On-SAC. Both cBC and rBC link to the key amacrine cells. The left side A17 and A2 (ac52-90) link onwards to W3a type ganglion cells, but not to DSGS’s. Also confirmed are central roles for the wide field amacrine cells A17, and the narrow field A2 and ac21-67. The latter are revealed as hosting the top signal flows in the present analysis. Both have strong links to cBC-1 to 4, as above, and also to cBC-6, 7, 8 and 9; suggesting a role in a Off-On detection circuit, which can be further investigated by these methods.

As noted by others [47], those neurons putatively identified as Horizontals cells [2], wherein they were called “other or orphan”) do not appear to have a prominent local role in the retinal fragment examined here. They may have a wider lateral spread of connections, beyond the 100 μm scale of the present retinal sample, and so are missed in the present analysis. Still, two of the top flow On-SAC’s are linked by one such Horizontal cell (cf. Fig 2).

The analysis developed herein provides one framework for analysing connectomic data and delineating link pathways that may be functioning circuits in the retina. That can only be verified by comparison with extant data probing such functions or, indeed, by further experiments. The sequence of figures presented illustrates how to probe the connectome and so reveal possible circuits. Further analysis, inspired by biologically relevant hypotheses, likely will reveal additional circuits.

## Supporting Information

S1 Data Weighted adjacency matrix for the 950 node mouse retina sample. (1.8 MB CSV text file).
(CSV)

S2 Data Module membership of the 950 nodes of the mouse retina. (2 KB text file).
(TXT)

S1 Fig Node degree distribution for the mouse retina sample. Log-Log histogram plot of the frequency of each weighted degree using the basic adjacency matrix (S1 Data) with cutoff: unity (0.16 μm^2^): filled blue circles; 10: O; 30: x. The reference line (red) has slope -2.
(TIF)

S2 Fig Node BC of each of the 950 nodes, calculated for the weighted network. (TIF)

S3 Fig SDE layout of high Degree nodes of the mouse retina network. 3D plot of the 950 neurons calculated using Spectral Distance Embedding (SDE) eigenvectors (y, z axes), with neuron types layered anatomically for clarity (x axis: BC at x = 0.1, to GC at x = 0.9). The top 10 cells, or network nodes, as ranked by weighted Degree (cf. S1 Table), are highlighted and labelled; along with their highest weight (>10) links. Neurons symbols are: rBC red squares, cBC red diamonds, H green triangle, WF ac light blue circles (SAC as triangle, pentagram), NFac dark blue circles, GC grey stars. Neurons are colored by type as in [2].
(TIF)

S4 Fig SDE layout using high node BC of the mouse retina network. 3D layout of the 950 neurons using SDE eigenvectors (y, z axes), with neuron types layered (x axis) as in S3 Fig. The top 10 cells, as ranked by weighted node BC (S2 Table), are highlighted and labelled; along with their highest weight (>10) links. Neurons symbols and colors as in S3 Fig.
(TIF)

S5 Fig 2D layout of cone BC modules from NG method. EM coordinates in the 2D retinal plane of key cone BCs coloured by their module membership, calculated by the Newman-Girvan method. Convex hull and centroid of each module is marked.
(TIF)

S6 Fig 3D plot of top flow SAC-Off cell and with its highest weight (>10) links to all other neurons. Cell positions, layers and module colors as in Fig 2. Scale bar, 10 μm.
(TIF)

S7 Fig 3D plot of top flow SAC-On cell and with its highest weight (>10) links to all other neurons. Cell positions, layers and module colors as in Fig 2. Scale bar, 10 μm.
(TIF)

S8 Fig 3D plot of top two SAC-On cells and with their highest weight (>10) links to all other neurons. Cell positions, layers and module colors as in Fig 2. Scale bar, 10 μm.
(TIF)

S9 Fig 3D plot of top flow amacrine cell and with its highest weight (>10) links to all other neurons. Cell positions, layers and module colors as in Fig 2. Scale bar, 10 μm.
(TIF)

S10 Fig 3D plot of second ranked amacrine cell and with its highest weight (>10) links to all other neurons. Cell positions, layers and module colors as in Fig 2. Scale bar, 10 μm.
(TIF)

S1 Table Top 10 nodes ranked by degree. Node degree of the mouse retina network, calculated from: A) the un-weighted adjacency matrix; and B) calculated from the weighted adjacency matrix. The node numerical ID and cell type are taken from the original data [2]. Degree listed in decreasing order.
(PDF)

S2 Table Top 10 node Betweeness Centrality. Node Betweeness Centrality (nBC) of the mouse retina network calculated from the weighted adjacency matrix, on the right The node numerical ID and cell type are taken from the original data ([2], S1 Data and [4]). nBC listed in decreasing order.
(PDF)

S3 Table Edge Betweeness Centrality (eBC) of the mouse retina network calculated from the weighted adjacency matrix for node pairs (i, j), using the Newman-Girvan [22] method. The nodes’ numerical IDs and cell types are taken from the original data ([2], S1 Data and [4]). eBC listed in decreasing order.
(PDF)

S4 Table Comparison of Module decomposition methods. Decomposition of the mouse retina network into modules as calculated by the methods indicated, showing the optimal number of modules and Newman’s modularity metric, Q. Trivial modules, containing one or only a few neurons, are excluded.
(PDF)

S5 Table Total signal Flow through selected nodes of the mouse retina network calculated using the InfoMap algorithm [25,28]. Total flow through all nodes sums to 1. The node numerical ID and cell type are taken from the original data [2]. Flows listed in decreasing order.
(PDF)

S6 Table Comparison of top nodes ranked by various measures. Top ranked nodes of the mouse retina network based on: node signal Flow fraction (InfoMap, cf Table 1), node betweenness Centrality (S2 Table) and weighted degree (S1 Table). Nodes identified by numerical ID [2], and listed in decreasing order.
(PDF)
