# Supplementary figures and images for: Network Analysis and Visualization of Mouse Retina Connectivity Data
Source: PLoS One. 2016 Jul 14;11(7):e0158626. doi: 10.1371/journal.pone.0158626 (PMC4944929; doi:10.1371/journal.pone.0158626)

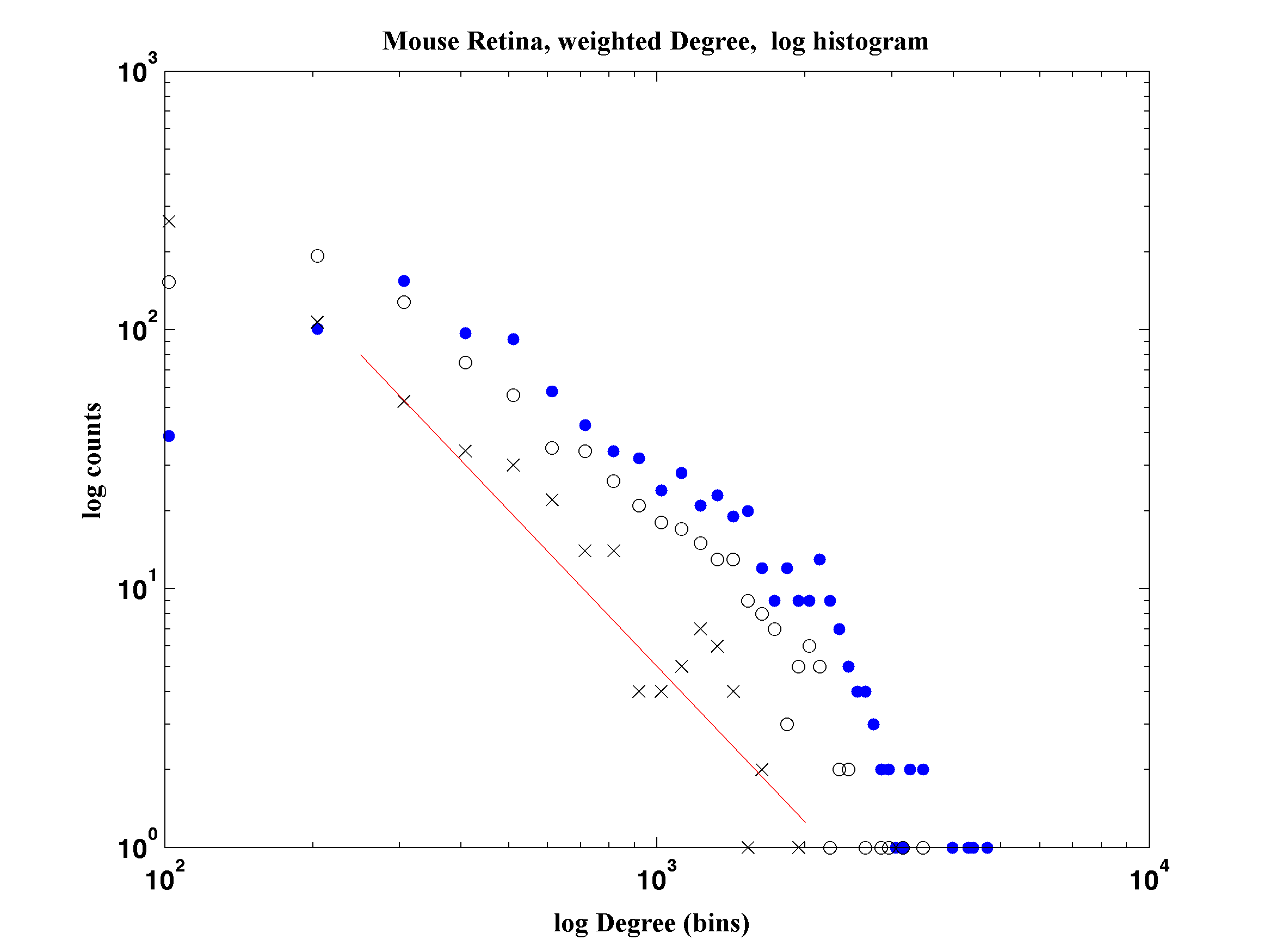

Supplement: S1 Fig — Log-Log histogram plot of the frequency of each weighted degree using the basic adjacency matrix (S1 Data) with cutoff: unity (0.16 μm2): filled blue circles; 10: O; 30: x. The reference line (red) has slope -2. (TIF) [file pone.0158626.s003.tif]

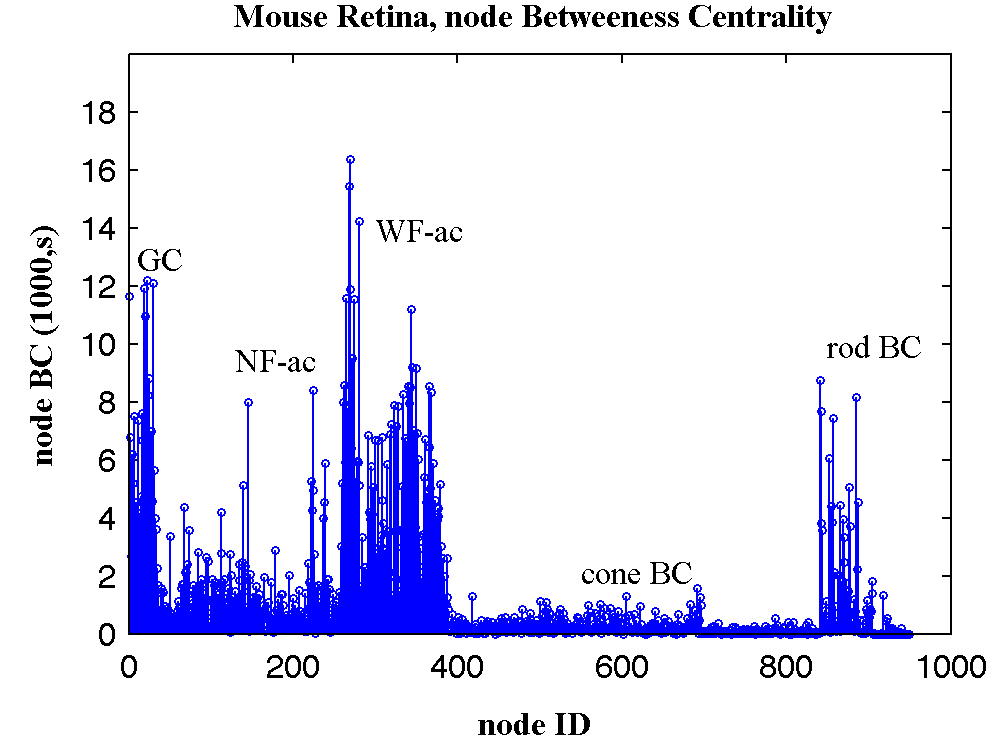

Supplement: S2 Fig — (TIF) [file pone.0158626.s004.tif]

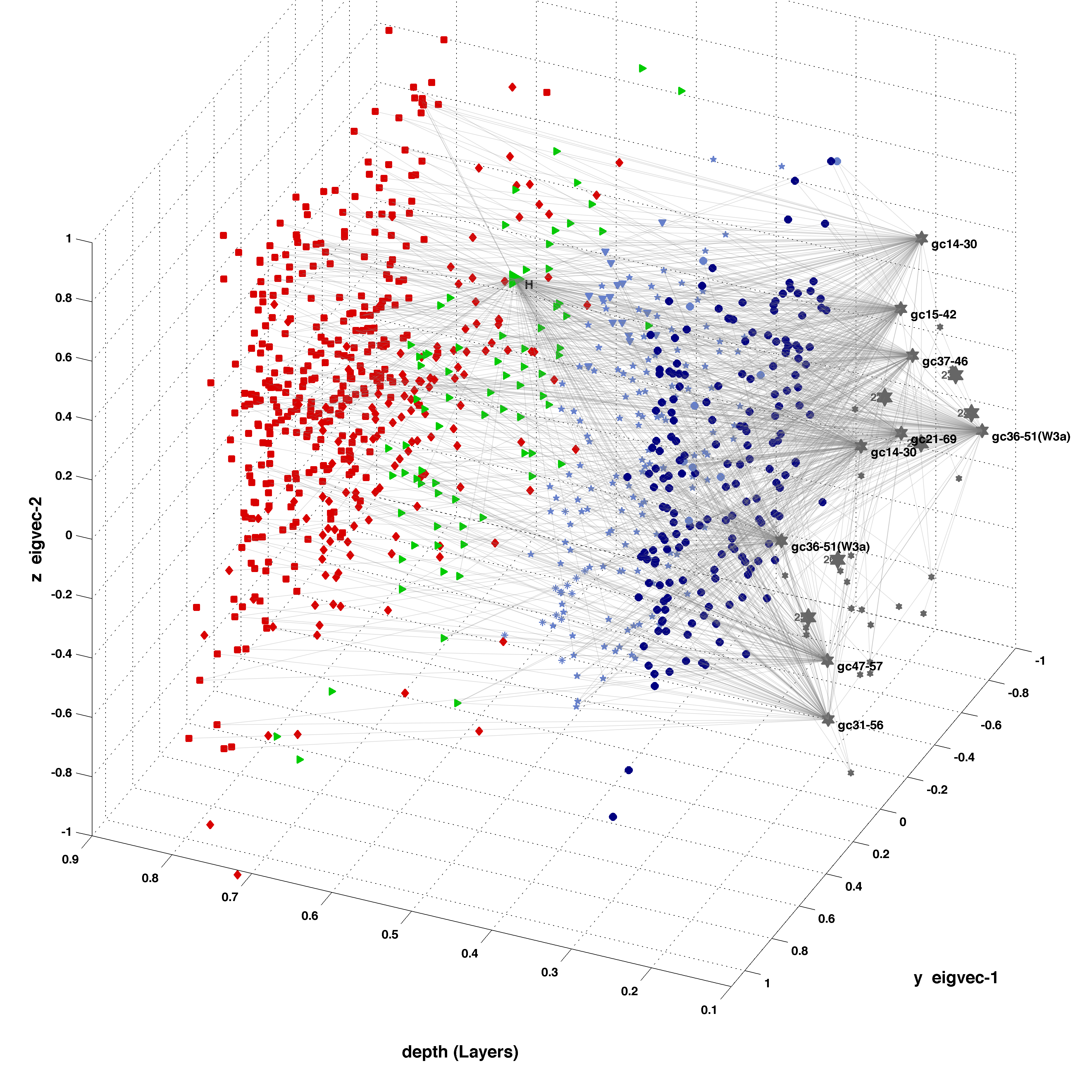

Supplement: S3 Fig — 3D plot of the 950 neurons calculated using Spectral Distance Embedding (SDE) eigenvectors (y, z axes), with neuron types layered anatomically for clarity (x axis: BC at x = 0.1, to GC at x = 0.9). The top 10 cells, or network nodes, as ranked by weighted Degree (cf. S1 Table), are highlighted and labelled; along with their highest weight (>10) links. Neurons symbols are: rBC red squares, cBC red diamonds, H green triangle, WF ac light blue circles (SAC as triangle, pentagram), NFac dark blue circles, GC grey stars. Neurons are colored by type as in [2]. (TIF) [file pone.0158626.s005.tif]

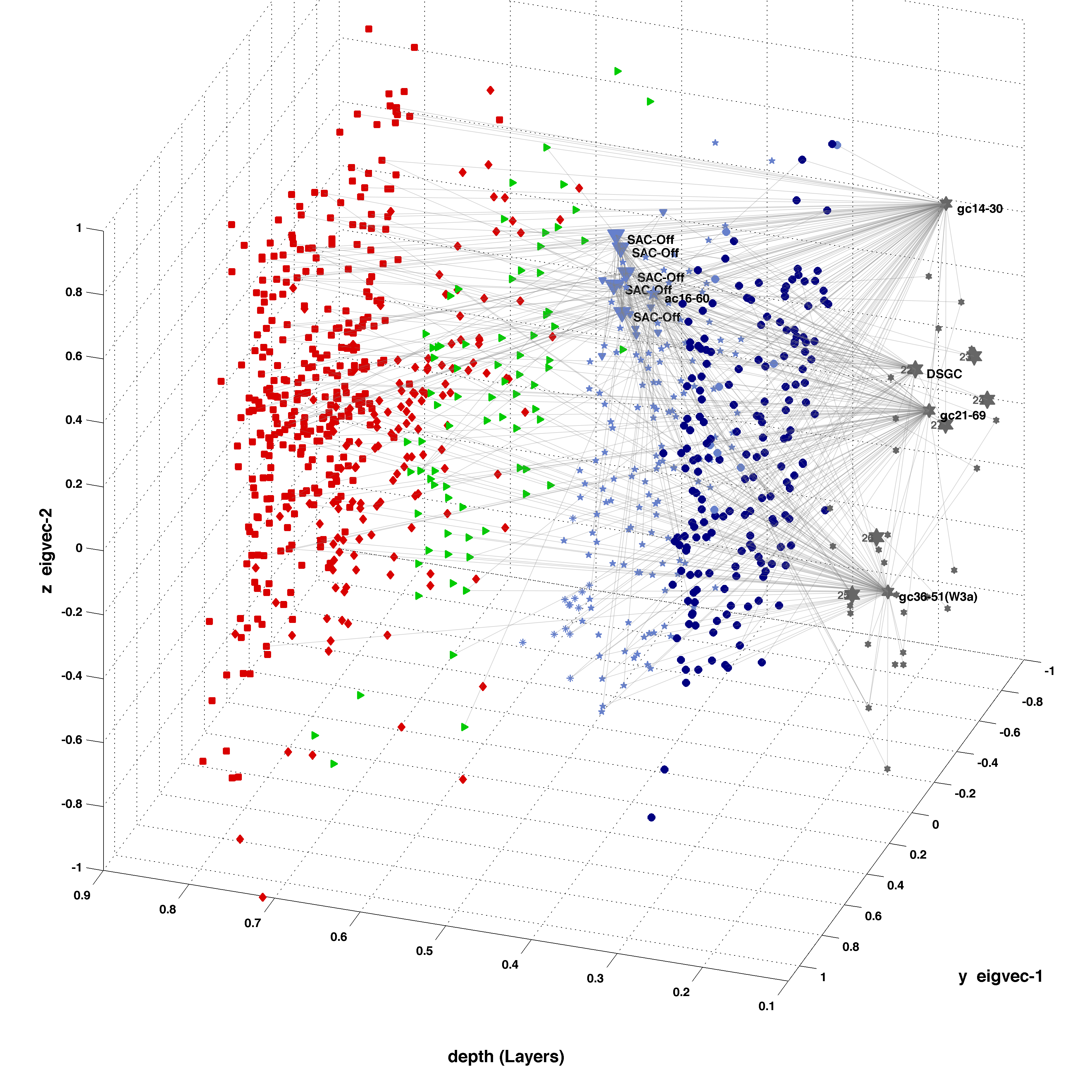

Supplement: S4 Fig — 3D layout of the 950 neurons using SDE eigenvectors (y, z axes), with neuron types layered (x axis) as in S3 Fig. The top 10 cells, as ranked by weighted node BC (S2 Table), are highlighted and labelled; along with their highest weight (>10) links. Neurons symbols and colors as in S3 Fig. (TIF) [file pone.0158626.s006.tif]

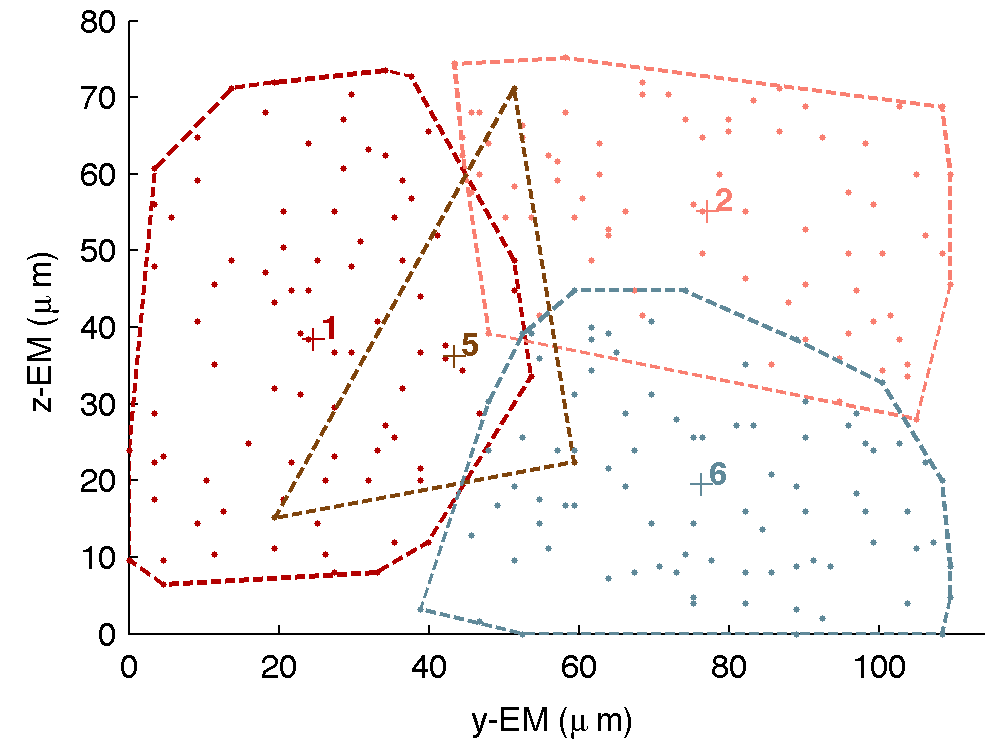

Supplement: S5 Fig — EM coordinates in the 2D retinal plane of key cone BCs coloured by their module membership, calculated by the Newman-Girvan method. Convex hull and centroid of each module is marked. (TIF) [file pone.0158626.s007.tif]

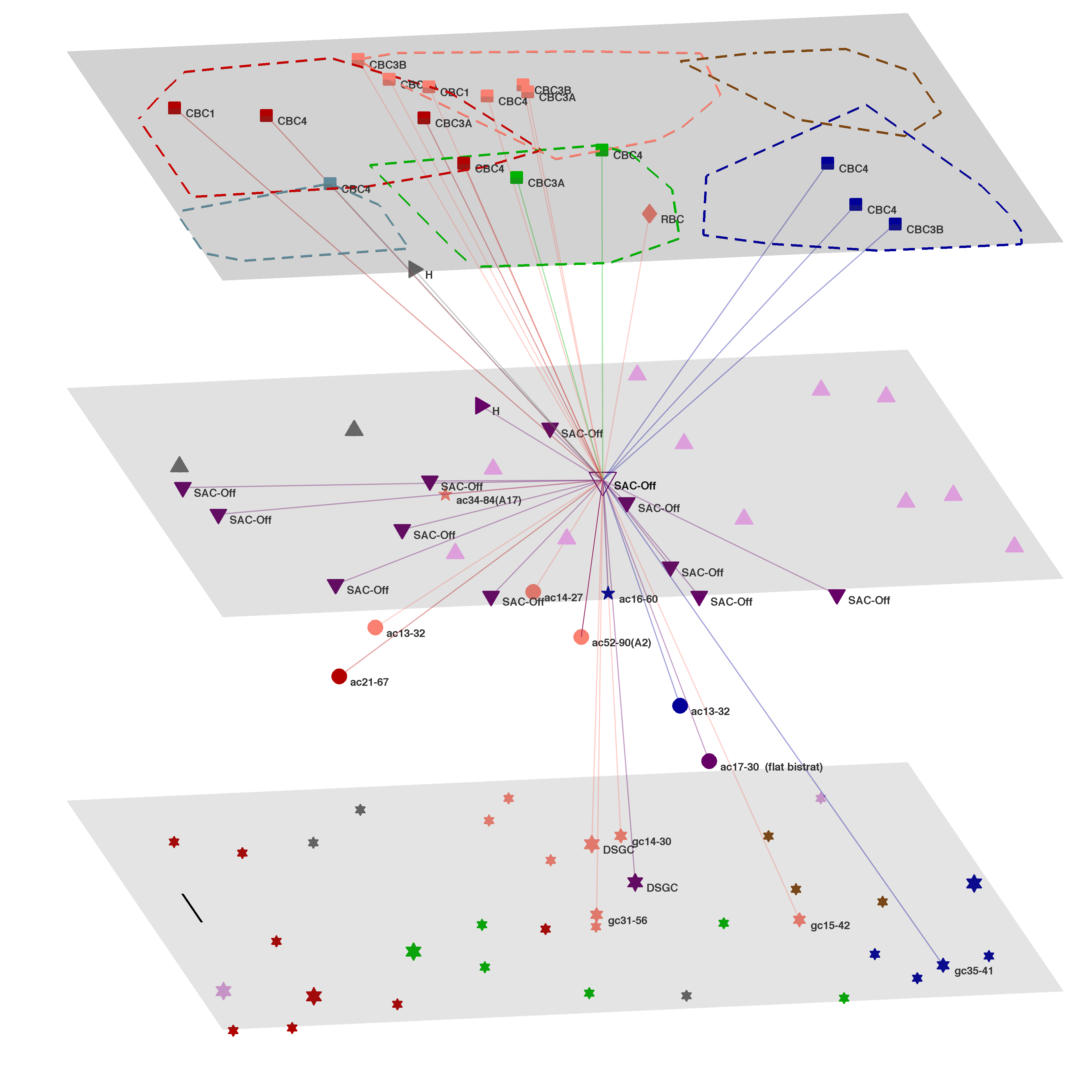

Supplement: S6 Fig — Cell positions, layers and module colors as in Fig 2. Scale bar, 10 μm. (TIF) [file pone.0158626.s008.tif]

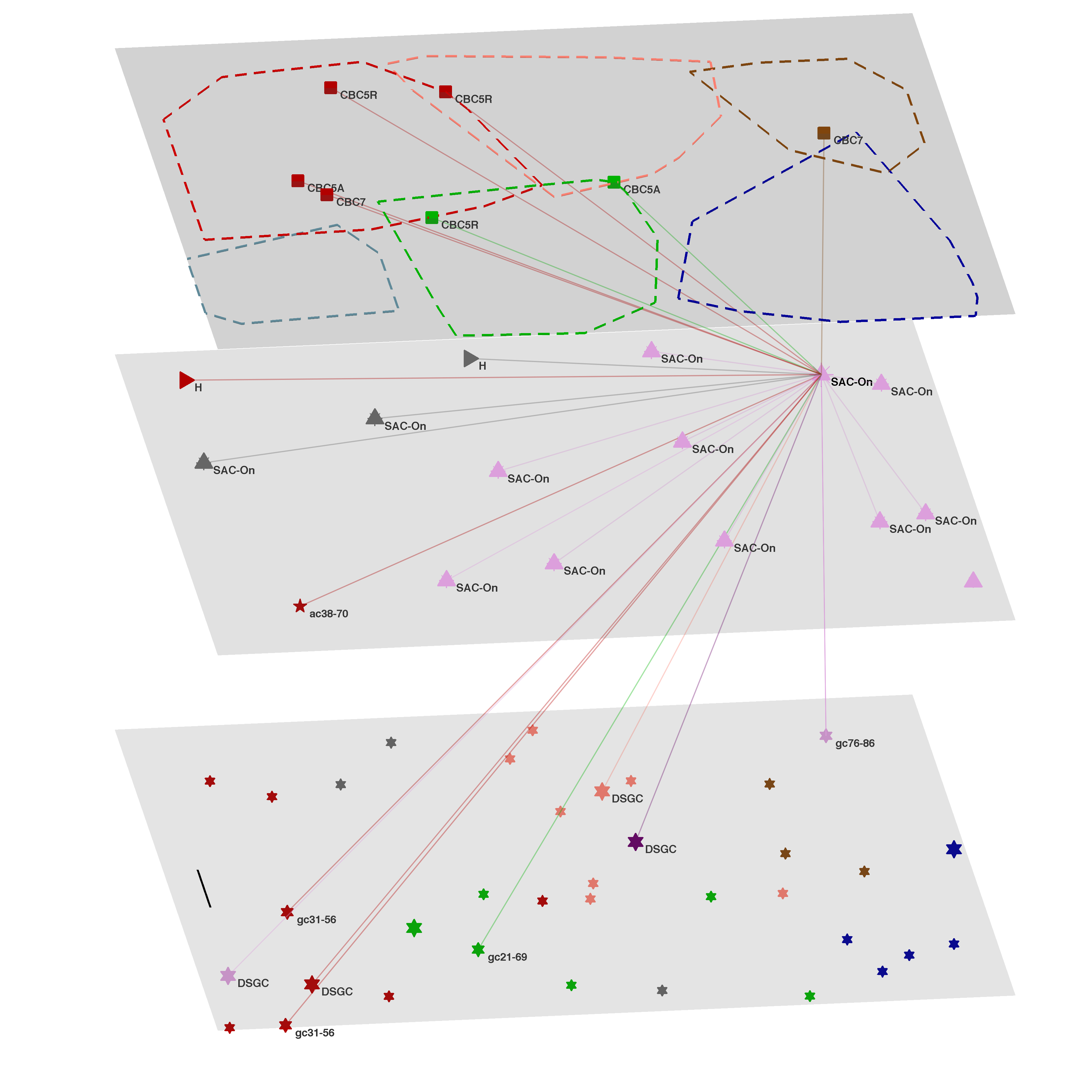

Supplement: S7 Fig — Cell positions, layers and module colors as in Fig 2. Scale bar, 10 μm. (TIF) [file pone.0158626.s009.tif]

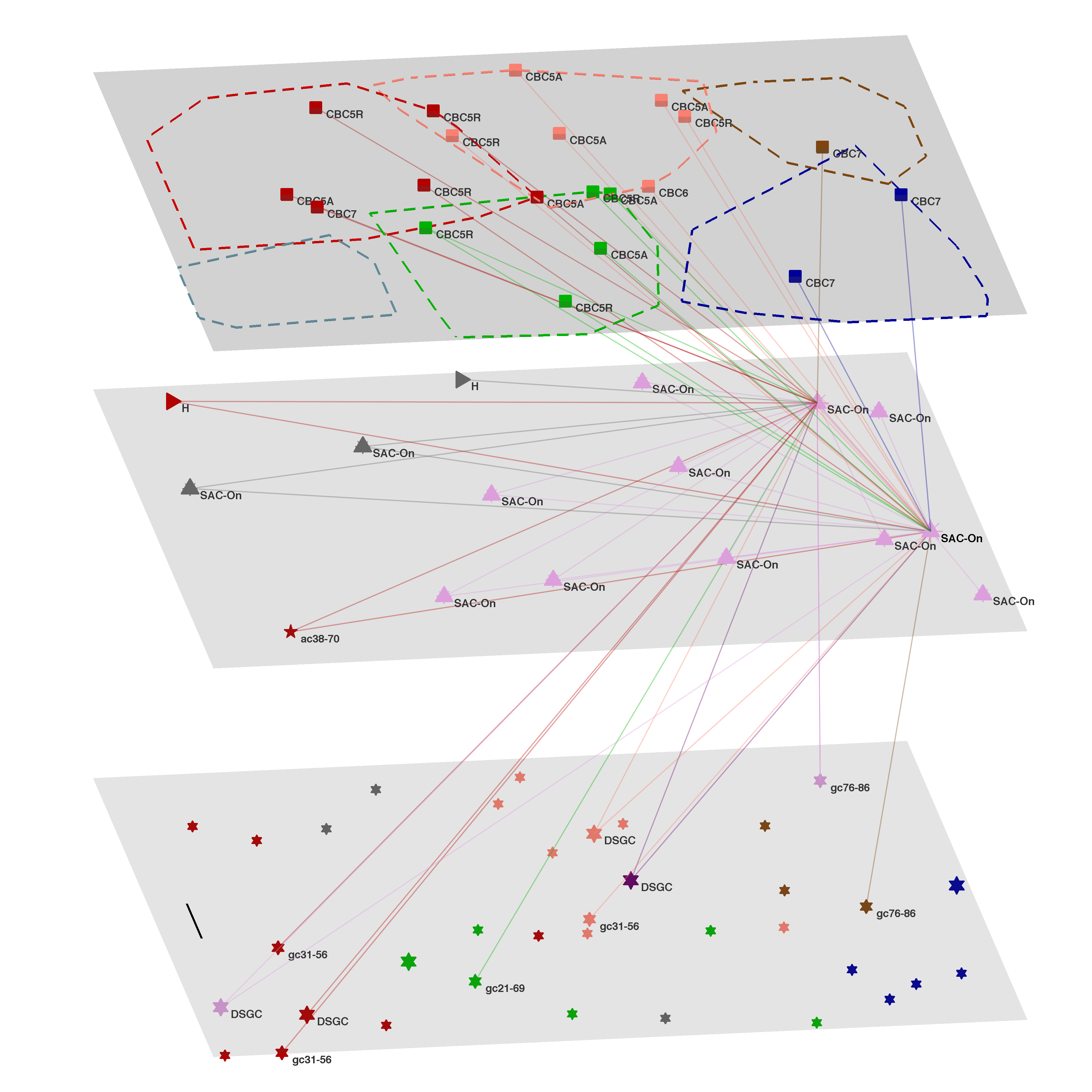

Supplement: S8 Fig — Cell positions, layers and module colors as in Fig 2. Scale bar, 10 μm. (TIF) [file pone.0158626.s010.tif]

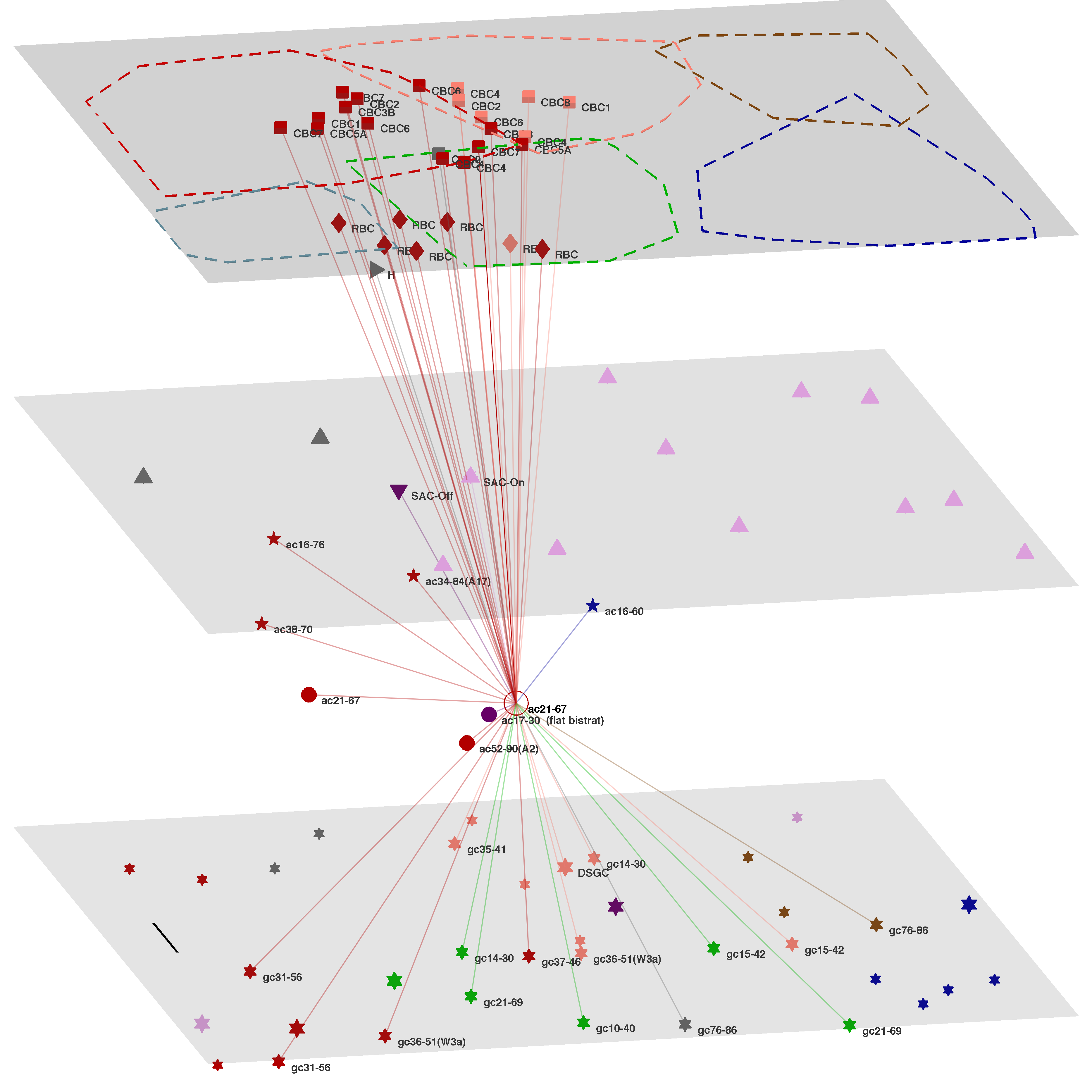

Supplement: S9 Fig — Cell positions, layers and module colors as in Fig 2. Scale bar, 10 μm. (TIF) [file pone.0158626.s011.tif]

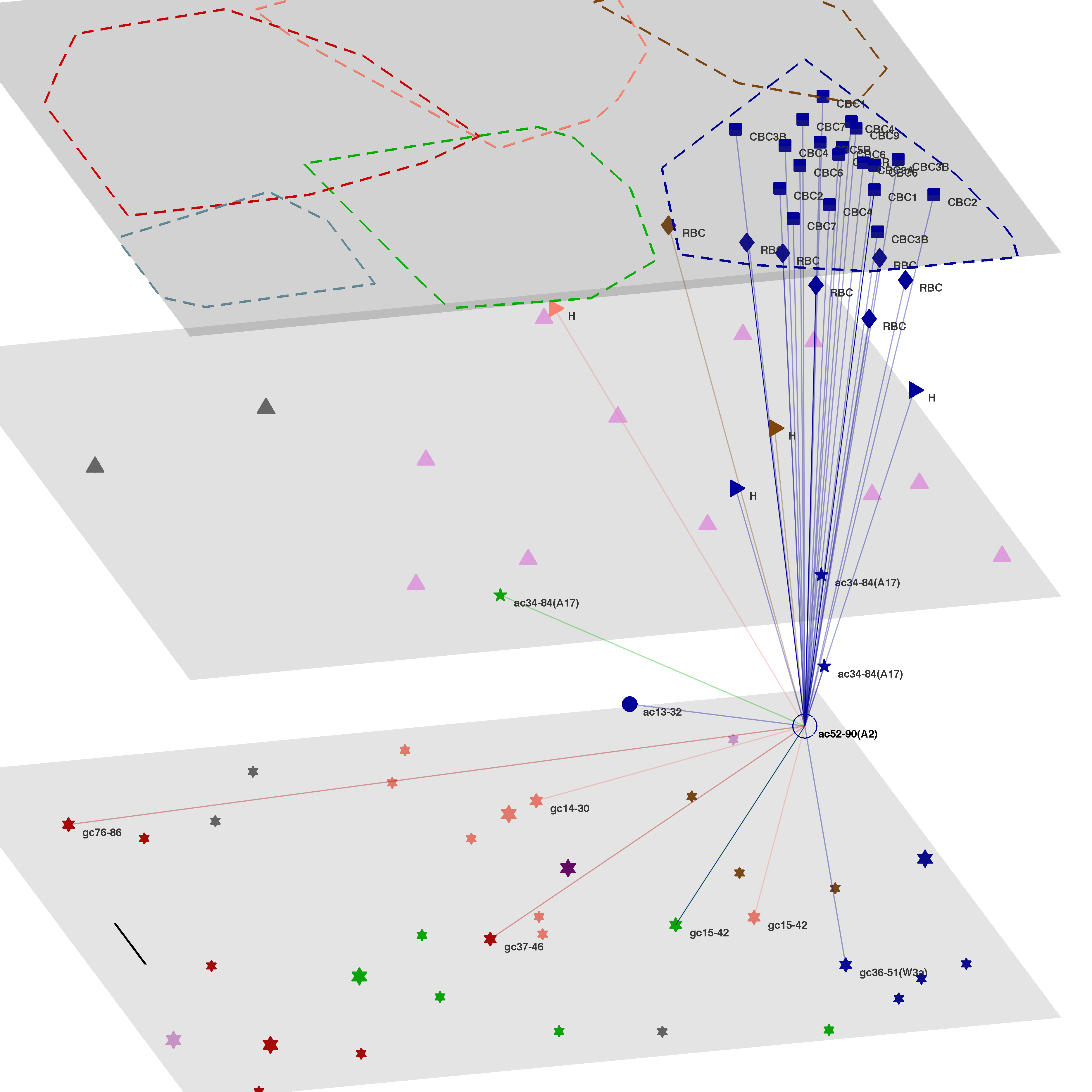

Supplement: S10 Fig — Cell positions, layers and module colors as in Fig 2. Scale bar, 10 μm. (TIF) [file pone.0158626.s012.tif]
