# Supplementary material for: Network Analysis and Visualization of Mouse Retina Connectivity Data
Source: PLoS One. 2016 Jul 14;11(7):e0158626. doi: 10.1371/journal.pone.0158626 (PMC4944929; doi:10.1371/journal.pone.0158626)
Supplement: S1 Table — Node degree of the mouse retina network, calculated from: A) the un-weighted adjacency matrix; and B) calculated from the weighted adjacency matrix. The node numerical ID and cell type are taken from the original data [2]. Degree listed in decreasing order. (PDF) [file pone.0158626.s013.pdf]

Table S1. Top 10 node Degrees.

A.

Node ID	Cell Type	Degree un-weighted
842	H	488
19	gc21-69	451
11	gc37-46	434
31	gc47-57	428
281	ac16-60	418
6	gc15-42	416
2	gc14-30	412
1	gc14-30	408
300	ac16-76	395

B.

Node ID	Cell Type	Degree weighted
6	gc15-42	4701
31	gc47-57	4402
842	H	4308
17	gc36 51(W3a)	3984
2	gc14-30	3493
16	gc36-51(W3a)	3470
19	gc21-69	3327
11	gc37-46	3273
1	gc14-30	3229
