# Supplementary material for: Network Analysis and Visualization of Mouse Retina Connectivity Data
Source: PLoS One. 2016 Jul 14;11(7):e0158626. doi: 10.1371/journal.pone.0158626 (PMC4944929; doi:10.1371/journal.pone.0158626)
Supplement: S2 Table — Node Betweeness Centrality (nBC) of the mouse retina network calculated from the weighted adjacency matrix, on the right The node numerical ID and cell type are taken from the original data ([2], S1 Data and [4]). nBC listed in decreasing order. (PDF) [file pone.0158626.s014.pdf]

Table S2. Top 10 node Betweeness Centrality.

Node ID	Cell type	nBC
270	SAC-Off	16358
268	SAC-Off	15439
281	ac16-60	14208
22	DSGC	12202
30	gc36-51(W3a)	12099
19	gc21-69	11913
269	SAC-Off	11896
1	gc14-30	11633
264	SAC-Off	11583
