# Supplementary material for: Network Analysis and Visualization of Mouse Retina Connectivity Data
Source: PLoS One. 2016 Jul 14;11(7):e0158626. doi: 10.1371/journal.pone.0158626 (PMC4944929; doi:10.1371/journal.pone.0158626)
Supplement: S3 Table — The nodes’ numerical IDs and cell types are taken from the original data ([2], S1 Data and [4]). eBC listed in decreasing order. (PDF) [file pone.0158626.s015.pdf]

Table S3. Top 10 edge Betweeness Centrality.

Node i	Node j	Cell Type (i)	Cell Type (j)	eBC
281	727	ac16-60	rBC	524
336	793	A17, ac34-84	rBC	384
337	806	A17, ac34-84	rBC	348
352	793	A17, ac34-84	rBC	267
15	825	gc31-56	rBC	237
275	806	Ac06-60	rBC	261
266	936	SAC-Off	H	245
326	896	ac38-70	H	234
1	758	gc14-30	rBC	219
