# Supplementary material for: Network Analysis and Visualization of Mouse Retina Connectivity Data
Source: PLoS One. 2016 Jul 14;11(7):e0158626. doi: 10.1371/journal.pone.0158626 (PMC4944929; doi:10.1371/journal.pone.0158626)
Supplement: S4 Table — Decomposition of the mouse retina network into modules as calculated by the methods indicated, showing the optimal number of modules and Newman’s modularity metric, Q. Trivial modules, containing one or only a few neurons, are excluded. (PDF) [file pone.0158626.s016.pdf]

Table S4. Comparison of Module decomposition methods.

Method	Number of Modules	Q
k-means	5	0.19
Fast Newman	6	0.22
InfoMap	8	0.26
Newman B matrix	5	0.15
