# Supplementary material for: Network Analysis and Visualization of Mouse Retina Connectivity Data
Source: PLoS One. 2016 Jul 14;11(7):e0158626. doi: 10.1371/journal.pone.0158626 (PMC4944929; doi:10.1371/journal.pone.0158626)
Supplement: S5 Table — Total flow through all nodes sums to 1. The node numerical ID and cell type are taken from the original data [2]. Flows listed in decreasing order. (PDF) [file pone.0158626.s017.pdf]

Table S5. Top 10 node signal Flows.

Node ID	Cell type	Flow fraction
120	ac21-67	0.0041
202	A2, ac52-90	0.00395
323	ac26-78	0.0039
351	A17, ac34-84	0.0038
328	ac38-70	0.0037
127	ac21-67	0.0037
300	ac42-50	0.0036
201	A2, ac52-90	0.0035
343	A17, ac34-84	0.0035
340	A17, ac34-84	0.0034
