# Supplementary material for: Network Analysis and Visualization of Mouse Retina Connectivity Data
Source: PLoS One. 2016 Jul 14;11(7):e0158626. doi: 10.1371/journal.pone.0158626 (PMC4944929; doi:10.1371/journal.pone.0158626)
Supplement: S6 Table — Top ranked nodes of the mouse retina network based on: node signal Flow fraction (InfoMap, cf Table 1), node betweenness Centrality (S2 Table) and weighted degree (S1 Table). Nodes identified by numerical ID [2], and listed in decreasing order. (PDF) [file pone.0158626.s018.pdf]

Table S6. Comparison of top nodes ranked by various measures.

Node ID		
InfoMap	Node BC	Degree (wt)
120	270	6
202	268	31
323	281	842
351	22	17
328	30	2
127	19	16
300	269	19
343	1	11
340	264	1
201	270	6
